# Supplementary figures and images for: A high-throughput cigarette smoke-treated bronchosphere model for disease-relevant phenotypic compound screening
Source: PLoS One. 2023 Jun 29;18(6):e0287809. doi: 10.1371/journal.pone.0287809 (PMC10310037; doi:10.1371/journal.pone.0287809)

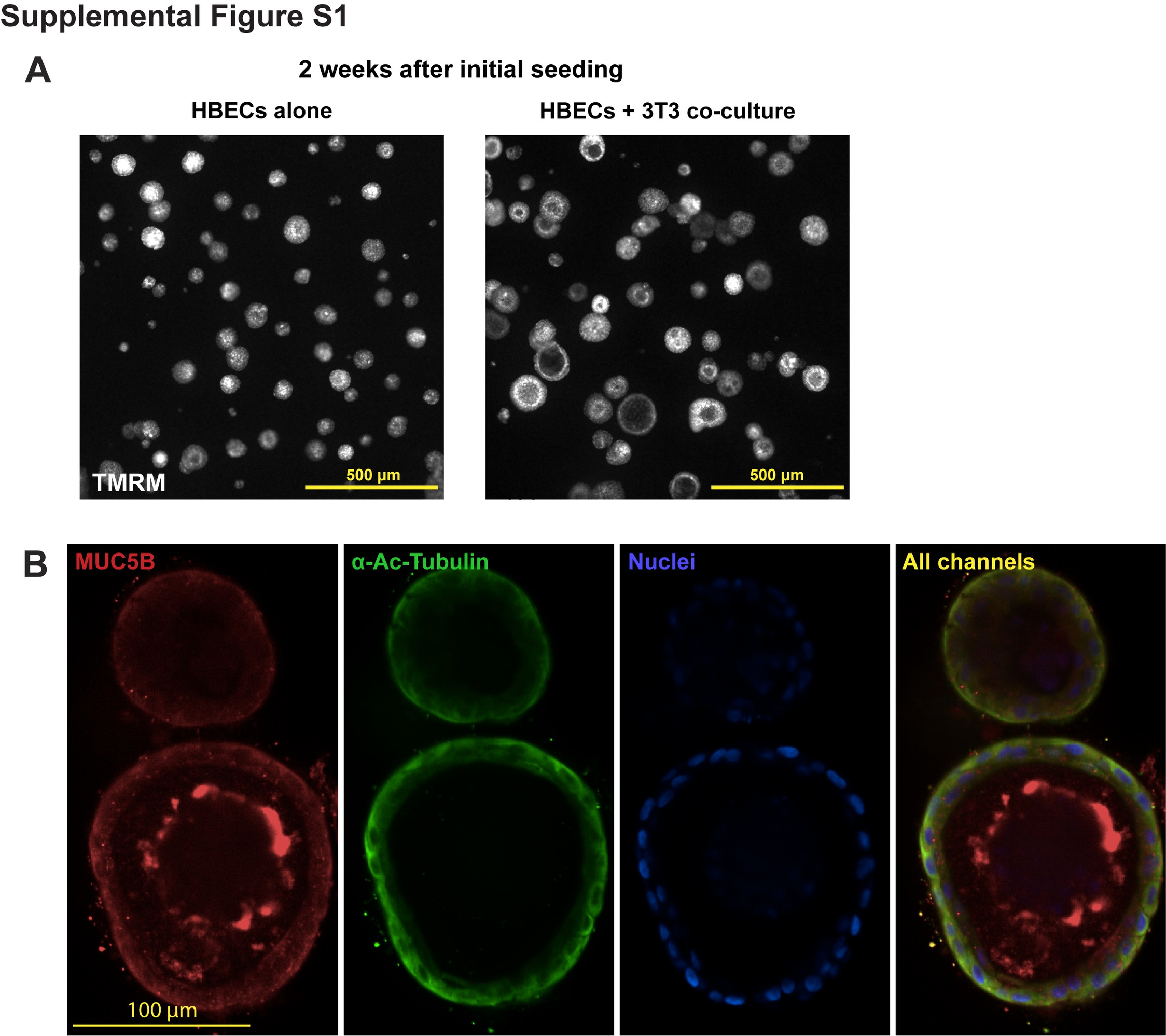

Supplement: S1 Fig — A) Bronchospheres 2 weeks after initial seeding, with and without 3T3 feeder layer. B) After two weeks, bronchospheres stain positive for MUC5B and α-acetylated tubulin, indicating that they have differentiated to contain secretory and ciliated cell subtypes, as previously shown(18,24). (TIF) [file pone.0287809.s001.tif]

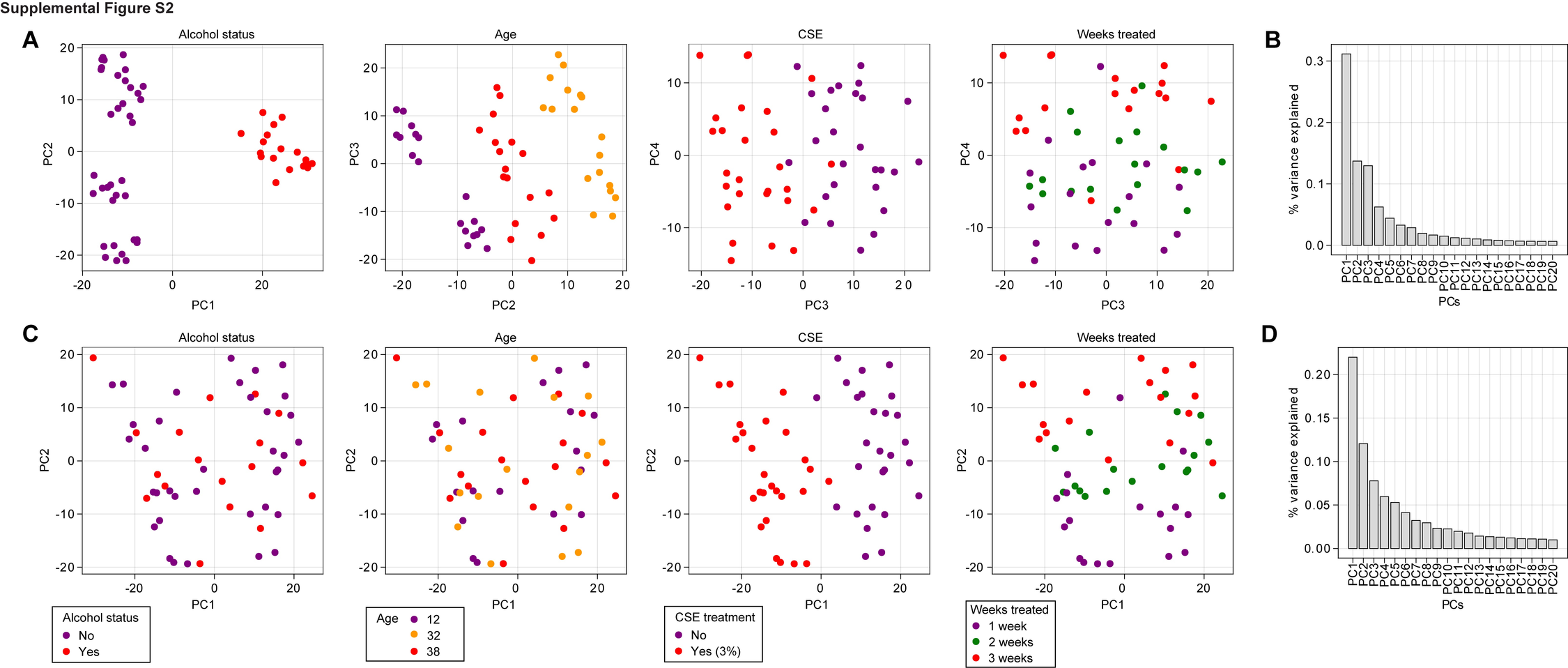

Supplement: S2 Fig — A) Scatter plots visualizing PC effects after DESeq2 normalization only. B) Bar plot describing the % of variance explained by each PC after DESeq2 normalization only. The major PCs capture donor specific effects, meaning that the most variance in the data is not reflective of CSE treatment effect. C) Scatter plots visualizing PC effects after removing the donor, age, and alcohol status using mixed model. The major PCs capture CSE treatment effect. D) Bar plot describing the % of variance explained by each PC after covariate adjustment using mixed model. (TIF) [file pone.0287809.s002.tif]

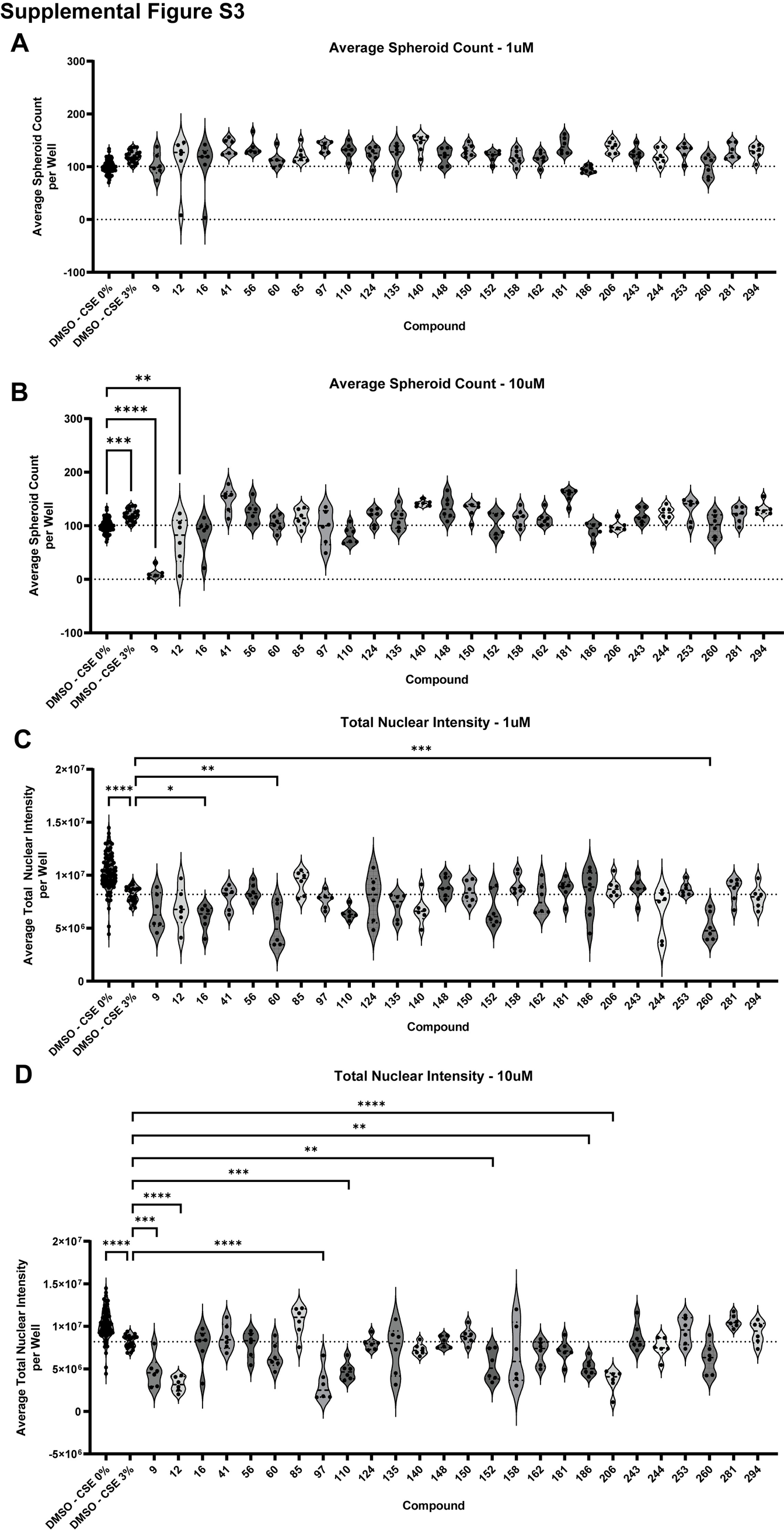

Supplement: S3 Fig — Compounds that were identified in the primary screen as modulators of both phenotypes were tested at 1μM and 10μM. The number of spheroids per well (spheroid counts) was measured from the spheroid size/swell readout with TMRM live dye staining, while the total nuclear intensity per well (total nuclear intensity) was measured from the MUC5AC ratio readout with Hoechst staining after fixation. Spheroid counts per well were calculated for compound treatment at A) 1μM and B) 10μM concentrations. Total nuclear intensity per well was calculated for compound treatment at C) 1μM and D) 10μM concentrations. Compounds that were found to significantly decrease counts or nuclear intensity were considered toxic and filtered out of final hit selection. They are indicated appropriately in Fig 4. All individual data points represent biological replicates. All plots were analyzed by ordinary one-way ANOVA with Dunnett’s multiple comparisons test. *p<0.05; **p<0.01, **p<0.01, ***p<0.001, ****p<0.0001. (TIF) [file pone.0287809.s003.tif]

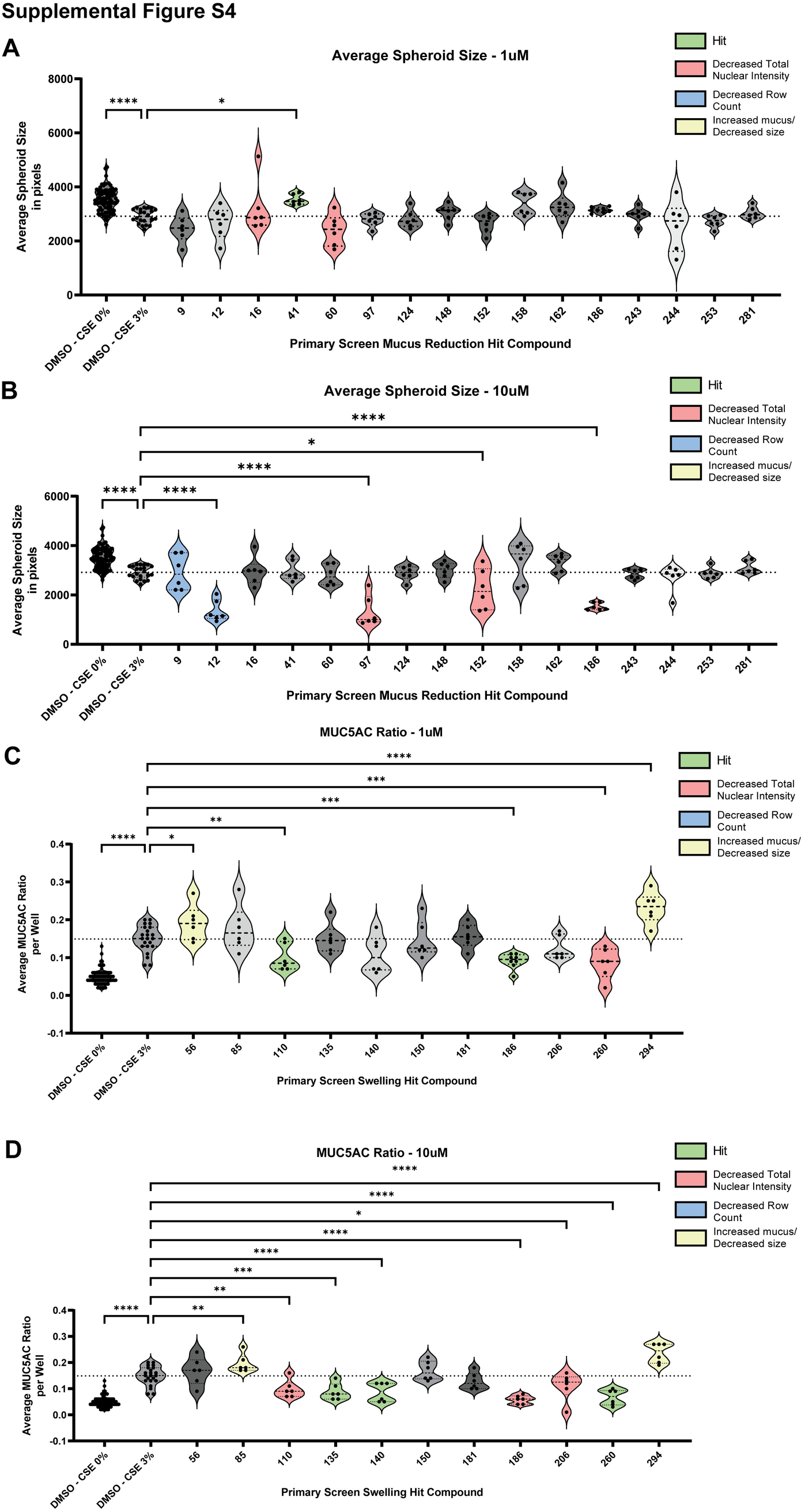

Supplement: S4 Fig — Compounds that were identified in the primary screen as modulators of MUC5AC reduction were tested at A) 1μM and B) 10μM concentrations to see if they affect spheroid size. No compounds caused significant shrinkage that were not already identified through the toxicity readouts in Fig 3. Compounds that were identified in the primary screen as modulators of spheroid size/swell were tested at C) 1μM and D) 10μM concentrations to see if they affect MUC5AC ratio. Compound 186 (1μM) is the only compound that was a primary hit in both swell and MUC5AC ratio and is plotted in both readouts in both Fig 4 and is identified as a hit compound in Fig 4C. Compounds 56 (1μM), 85 (10μM), and 294 (1 and 10μM) significantly increased MUC5AC ratio compared to DMSO + CSE 3% control and were therefore filtered out of swell hit selection. These compounds are appropriately labeled in Fig 4. Hits (colored green) identified in A)-D) were not selected as final hits since they did not produce the desired effect in the primary screen (Fig 3). All individual data points represent biological replicates. All plots were analyzed by ordinary one-way ANOVA with Dunnett’s multiple comparisons test. *p<0.05; **p<0.01, **p<0.01, ***p<0.001, ****p<0.0001. (TIF) [file pone.0287809.s004.tif]

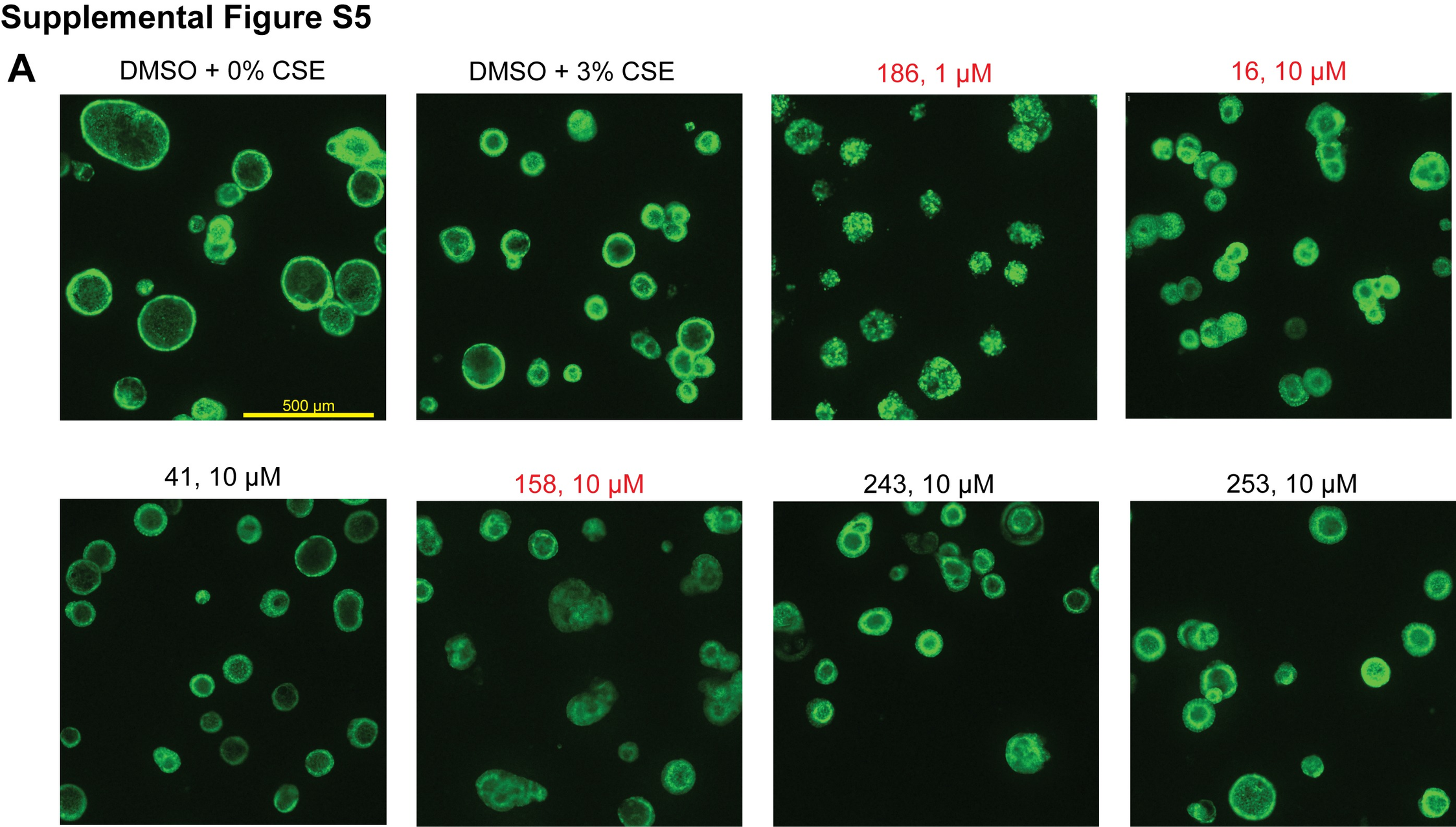

Supplement: S5 Fig — A) TMRM live dye-stained images of bronchospheres treated with hit compounds and concentrations. The images reveal that Compound 186 (1μM), 16 (10μM), and 158 (10μM) cause phenotypic changes to the bronchospheres that indicate an unhealthy state compared to DMSO + 3% CSE control, such as rough spheroid boundaries or loss of a clear lumen (indicated in red). These compounds have thus been filtered out of the final hit list. (TIF) [file pone.0287809.s005.tif]

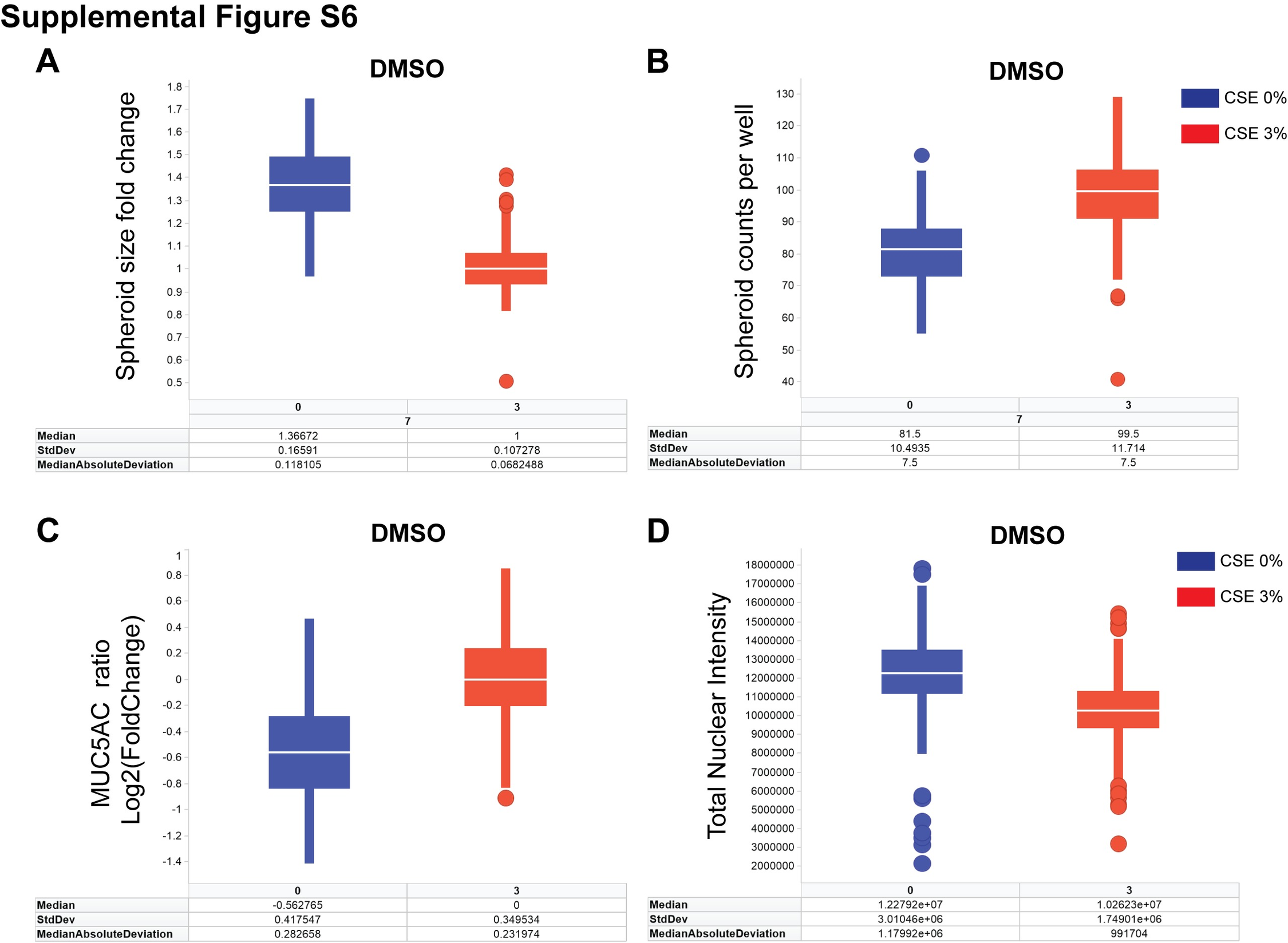

Supplement: S6 Fig — DMSO-treated spheroids at both 0 and 3% CSE were plotted after plate normalization (see details in “Primary compound screen and hit analysis” section of Methods) to obtain the median absolute deviation (MAD) for A) the spheroid size fold change and B) the spheroid counts per well from the spheroid size assay, and C) the MUC5AC ratio Log2(Fold Change) and D) the total nuclear intensity from the MUC5AC reduction assay. (TIF) [file pone.0287809.s006.tif]
